# Statement of Retraction: Graphene based scaffolds on bone tissue engineering

**DOI:** 10.1080/21655979.2025.2491936

**Published:** 2025-04-15

We, the Editor and Publisher of *Bioengineered*, have retracted the following article:

Nasrin Shadjou, Mohammad Hasanzadeh & Balal Khalilzadeh (2018) Graphene based scaffolds on bone tissue engineering, Bioengineered, 9:1, 38-47, DOI: 10.1080/21655979.2017.1373539

Following publication, the journal was made aware by a third party that the published article contains unreferenced similarity with the following published article:

- Amini AR, Laurencin CT, Nukavarapu SP. Bone tissue engineering: recent advances and challenges. Crit Rev Biomed Eng. 2012;40(5):363-408. https://doi.org/10.1615/critrevbiomedeng.v40.i5.10

Upon further review, it was identified that there was additional unreferenced similarity to the following published article:

- Rey C, Combes C, Drouet C, Sfihi H, Barroug A. Physico-chemical properties of nanocrystalline apatites: Implications for biominerals and biomaterials. Mat Sci Eng C. 2007;27(2):198-205. https://doi.org/10.1016/j.msec.2006.05.015

In addition to this, whilst they are referenced, there is similarity with the following published articles:

- Ma J and Qin J. Graphene-like Zinc Substituted Hydroxyapatite. Cryst. Growth Des. 2015;15(3):1273–1279. https://pubs.acs.org/doi/10.1021/cg501659x
- Gao C, Liu T, Shuai C, Peng S. Enhancement mechanisms of graphene in nano-58S bioactive glass scaffold: mechanical and biological performance. Sci Rep. 2014;4:4712. https://doi.org/10.1038/srep04712

When approached for an explanation, the authors have not provided an explanation for the similarity. As we cannot verify the validity of the published work or that it complied with our editorial policies, we are therefore retracting the article. The authors listed in this publication have been informed.

We have been informed in our decision-making by our editorial policies and the COPE guidelines. The retracted article will remain online to maintain the scholarly record, but it will be digitally watermarked on each page as ‘Retracted’. 

